# Diverse roles of FUS in Amyotrophic Lateral Sclerosis

**DOI:** 10.1186/2193-1801-4-S1-L54

**Published:** 2015-06-12

**Authors:** Maria Teresa Carri, Francesca Bozzo, Illari Salvatori, Cristiana Valle

**Affiliations:** University of Rome Tor Vergata, Rome, Italy; Fondazione Santa Lucia, Rome, Italy; Institute of Cell Biology and Neurobiology, CNR, Rome, Italy

**Keywords:** ALS, protein aggregation, mitochondrial damage

A number of different genes have been found mutated in patients with Amyotrophic Lateral Sclerosis (ALS). Several of these genes encode for proteins involved in multiple steps of RNA processing, suggesting that mRNA dys-metabolism has a role in the degeneration of motor neurons. This is the case also for FUS-linked ALS. FUS (Fused in Sarcoma) is a DNA/RNA binding proteins with an established, yet not completely clear, role in the regulation of RNA transcription, splicing, transport and translation. However, recent evidence indicates that (similarly to mutant ALS-linked SOD1) the toxic function of this protein may lie also in its propensity to aggregate and sequester other proteins, and/or in its ability to induce mitochondrial damage and oxidative stress. In this presentation I will discuss our recent work on the molecular mechanisms underlying these effects and their potential relevance in the pathogenesis of ALS.

